# An international meta-analysis confirms the association of *BNC2* with adolescent idiopathic scoliosis

**DOI:** 10.1038/s41598-018-22552-x

**Published:** 2018-03-16

**Authors:** Yoji Ogura, Kazuki Takeda, Ikuyo Kou, Anas Khanshour, Anna Grauers, Hang Zhou, Gang Liu, Yan-Hui Fan, Taifeng Zhou, Zhihong Wu, Yohei Takahashi, Morio Matsumoto, Japan Scoliosis Clinical Research Group (JSCRG), Noriaki Kawakami, Taichi Tsuji, Koki Uno, Teppei Suzuki, Manabu Ito, Shohei Minami, Toshiaki Kotani, Tsuyoshi Sakuma, Haruhisa Yanagida, Hiroshi Taneichi, Ikuho Yonezawa, Hideki Sudo, Kazuhiro Chiba, Naobumi Hosogane, Kotaro Nishida, Kenichiro Kakutani, Tsutomu Akazawa, Takashi Kaito, Kei Watanabe, Katsumi Harimaya, Yuki Taniguchi, Hideki Shigematsu, Satoru Demura, Takahiro Iida, Katsuki Kono, Eijiro Okada, Nobuyuki Fujita, Mitsuru Yagi, Masaya Nakamura, Texas Scottish Rite Hospital for Children Clinical Group (TSRHCCG), Lori A. Karol, Karl E. Rathjen, Daniel J. Sucato, John G. Birch, Charles E. Johnston, Benjamin S. Richards, Brandon Ramo, Amy L. McIntosh, John A. Herring, Todd A. Milbrandt, Vishwas R. Talwakar, Henry J. Iwinski, Ryan D. Muchow, J. Channing Tassone, X. -C. Liu, Richard Shindell, William Schrader, Craig Eberson, Anthony Lapinsky, Randall Loder, Joseph Davey, Elisabet Einarsdottir, Juha Kere, Dongsheng Huang, Guixing Qiu, Leilei Xu, Yong Qiu, Carol A. Wise, You-Qiang Song, Nan Wu, Peiqiang Su, Paul Gerdhem, Kota Watanabe, Shiro Ikegawa

**Affiliations:** 1 Laboratory of Bone and Joint Diseases, Center for Integrative Medical Sciences, RIKEN, Tokyo, Japan; 2 Department of Orthopaedic Surgery, Keio University School of Medicine, Tokyo, Japan; 3 Sarah M. and Charles E. Seay Center for Musculoskeletal Research, Texas Scottish Rite Hospital for Children, Dallas, Texas USA; 4 Department of Orthopaedics, Sundsvall and Härnösand County Hospital, Sundsvall, Sweden; 5 Department of Clinical Science, Intervention and Technology (CLINTEC) Karolinska Institutet, Stockholm, Sweden; 6 Department of Orthopaedic Surgery, The First Affiliated Hospital of Sun Yat-Sen University, Guangzhou, China; 7 Department of Orthopedic Surgery, Peking Union Medical College Hospital, Peking Union Medical College and Chinese Academy of Medical Sciences, Beijing, China; 8 Department of Biochemistry, University of Hong Kong, Hong Kong, China; 9 Department of Central Laboratory, Peking Union Medical College Hospital, Peking Union Medical College & Chinese Academy of Medical Sciences, Beijing, China; 10 Beijing Key Laboratory for Genetic Research of Skeletal Deformity, Beijing, China; 11 Medical Research Center of Orthopedics, Chinese Academy of Medical Sciences, Beijing, China; 12 Folkhälsan Institute of Genetics, and Molecular Neurology Research Program, University of Helsinki, Helsinki, Finland; 13 Department of Biosciences and Nutrition, Karolinska Institutet, Huddinge, Sweden; 14 Department of Medical and Molecular Genetics, King’s College London, Guy’s Hospital, London, United Kingdom; 15 Department of Spine Surgery, The Sun Yat-Sen Memorial Hospital of Sun Yat-Sen University, Guangzhou, China; 16 Department of Spine Surgery, The Affiliated Drum Tower Hospital of Nanjing University Medical School, Nanjing, China; 17 McDermott Center for Human Growth and Development, Department of Pediatrics and Department of Orthopaedic Surgery, University of Texas Southwestern Medical Center at Dallas, Dallas, Texas USA; 18 Department of Orthopaedics, Karolinska University Hospital, Huddinge, Sweden; 19 Department of Orthopaedic Surgery, Meijo Hospital, Nagoya, Japan; 20 Department of Orthopaedic Surgery, National Hospital Organization, Kobe Medical Center, Kobe, Japan; 21 Department of Orthopaedic Surgery, National Hospital Organization, Hokkaido Medical Center, Sapporo, Japan; 22 Department of Orthopaedic Surgery, Seirei Sakura Citizen Hospital, Sakura, Japan; 23 Department of Orthopaedic Surgery, Fukuoka Children’s Hospital, Fukuoka, Japan; 24 Department of Orthopaedic Surgery, Dokkyo Medical University School of Medicine, Mibu, Japan; 25 Department of Orthopaedic Surgery, Juntendo University School of Medicine, Tokyo, Japan; 26 Department of Advanced Medicine for Spine and Spinal Cord Disorders, Hokkaido University Graduate School of Medicine, Sapporo, Japan; 27 Department of Orthopaedic Surgery, National Defense Medical College, Tokorozawa, Japan; 28 Department of Orthopaedic Surgery, Kobe University Graduate School of Medicine, Kobe, Japan; 29 Department of Orthopaedic Surgery, St. Marianna University School of Medicine, Kawasaki, Japan; 30 Department of Orthopaedic Surgery, Osaka University Graduate School of Medicine, Suita, Japan; 31 Department of Orthopaedic Surgery, Niigata University Hospital, Niigata, Japan; 32 Department of Orthopaedic Surgery, Graduate School of Medical Sciences, Kyushu University, Fukuoka, Japan; 33 Department of Orthopaedic Surgery, Faculty of Medicine, The University of Tokyo, Tokyo, Japan; 34 Department of Orthopaedic Surgery, Nara Medical University, Nara, Japan; 35 Department of Orthopaedic Surgery, Kanazawa University School of Medicine, Kanazawa, Japan; 36 Department of Orthopaedic Surgery, Dokkyo Medical University Koshigaya Hospital, Koshigaya, Japan; 37 Department of Orthopaedic Surgery, Kono Othopaedic Clinic, Tokyo, Japan; 38 Department of Orthopaedic Surgery, Texas Scottish Rite Hospital for Children, Dallas, Texas USA; 39 Department of Orthopaedic Surgery, Shriners Hospitals for Children, Lexington, Kentucky USA; 40 Department of Orthopaedic Surgery, Children’s Hospital of Wisconsin, Milwaukee, Wisconsin USA; 41 OrthoArizona, Phoenix, Arizona USA; 42 Departments of Orthopedics, Sports Medicine, and Surgical Services, Akron Children’s Hospital, Akron, Ohio, USA; 43 Pediatric Orthopaedics and Scoliosis, Hasbro Children’s Hospital, Providence, Rhode Island USA; 44 University of Massachusetts Memorial Medical Center, Worcester, Massachusetts, USA; 45 Indiana University-Purdue University Indianapolis, Indianapolis, Indiana, USA; 46 University of Oklahoma Health Sciences Center, Oklahoma City, Oklahoma USA

## Abstract

Adolescent idiopathic scoliosis (AIS) is a common spinal deformity with the prevalence of approximately 3%. We previously conducted a genome-wide association study (GWAS) using a Japanese cohort and identified a novel locus on chromosome 9p22.2. However, a replication study using multi-population cohorts has not been conducted. To confirm the association of 9p22.2 locus with AIS in multi-ethnic populations, we conducted international meta-analysis using eight cohorts. In total, we analyzed 8,756 cases and 27,822 controls. The analysis showed a convincing evidence of association between rs3904778 and AIS. Seven out of eight cohorts had significant *P* value, and remaining one cohort also had the same trend as the seven. The combined *P* was 3.28 × 10^−18^ (odds ratio = 1.19, 95% confidence interval = 1.14–1.24). *In silico* analyses suggested that *BNC2* is the AIS susceptibility gene in this locus.

## Introduction

Adolescent idiopathic scoliosis (AIS) is a complex, three-dimensional spinal deformity. AIS occurs in otherwise healthy children from the age of 10 to the end of growth^1^. AIS is a common disease, affecting 2–3% of children, predominantly girls^1^. Its pathogenesis has been unknown; however twin studies and heritability, in which estimated penetrance in at-risk males is approximately 9% and estimated penetrance in at-risk females is approximately 29%, suggest that genetic components play an important role in the onset of AIS^2,3^. In fact, genome-wide association studies (GWASs) have identified eight loci associated with AIS^4–9^.

Confirming the association of previously identified loci in other populations is quite important to identify susceptibility genes. For AIS loci, however, sufficient multi-population studies have not been conducted except for the *LBX1* locus on chromosome 10q24.31^10–12^. We previously identified that an AIS locus on chromosome 9p22.2 represented by rs3904778 and reported *BNC2* as a candidate susceptibility gene in the locus based on *in vitro* and *in vivo* functional analyses for its causality^6^. To confirm the association of the 9p22.2 locus and examine its significance in different ethnic populations, we recruited multi-ethnic populations, including Japanese, Han Chinese and Caucasian and conducted a meta-analysis of rs3904778. The results showed that the *BNC2* locus is related to risk of AIS globally.

## Results

### Association of rs3904778 and AIS susceptibility

**Figure 1:**
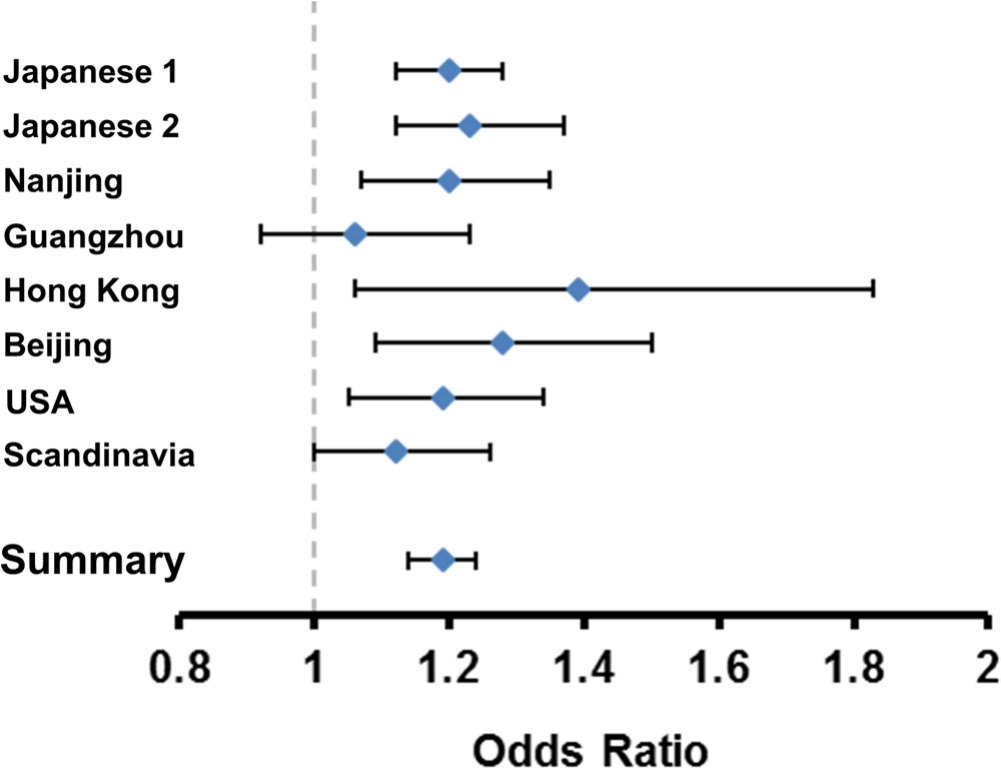
Forest plots for the association of rs3904778 with AIS susceptibility. The odds ratios and 95% confidence intervals were estimated based on the fixed-effect model. The contributing effect from each study is shown by a square with its confidence interval indicated by a horizontal line. Summary: the combined meta-analysis estimate.

We conducted the meta-analysis of rs3904778 using eight cohorts (Table 1). The data used for the analysis are presented in Supplementary Tables 1 and 2. They conformed to the Hardy-Weinberg disequilibrium (P > 1 × 10^−6^) and call rate of >99% as previously described quality control criteria^9^. We evaluated the association in each cohort using the Cochrane-Armitage trend test and and logistic regression. We combined the data using the inverse-variance method assuming a fixed-effects model. Three cohorts were previously reported^4,6^, and the other five were recruited for this study that included cohorts from Guangzhou, Hong Kong, Beijing, USA, and Scandinavia. For the GWAS cohorts, the possibility of population stratification has been evaluated and is unlikely (λs are all < 1.1)^4,6,9^. In total, 8,756 cases and 27,822 controls were included in the analysis, which showed a significant association: combined *P* = 3.28 × 10^−18^; odds ratio (OR) = 1.19; 95% confidence interval (CI) = 1.14–1.24 (Table 1). ORs were >1 in all eight cohorts, with little difference between ethnic groups according to the Forrest plot (Fig. 1). The analysis did not show any significant heterogeneity (Table 1), suggesting no statistical difference between studies.

**Table 1:** Association of rs3904778 with adolescent idiopathic scoliosis.

Population	Study	Number of samples		RAF		*P* value	Odds ratio (95% CI)	P_het_
		Case	Control	Case	Control			
Japanese	Japanese 1	2,109	11,140	0.459	0.413	2.10 × 10^−7^	1.20 (1.12–1.28)	
	Japanese 2	955	3,551	0.476	0.424	4.46 × 10^−5^	1.23 (1.12–1.37)	
	Japanese combined	3,064	14,691			5.08 × 10^−11^	1.21 (1.15–1.28)	0.68
Chinese	Nanjing	1,268	1,173	0.429	0.384	1.14 × 10^−3^	1.20 (1.07–1.35)	
	Guangzhou	659	1,063	0.354	0.340	3.77 × 10^−1^	1.06 (0.92–1.23)	
	Hong Kong	193	294	0.380	0.306	1.90 × 10^−2^	1.39 (1.06–1.83)	
	Beijing	480	861	0.457	0.397	2.50 × 10^−3^	1.28 (1.09–1.50)	
	Chinese combined	2,600	3,391			6.07 × 10^−6^	1.19 (1.10–1.28)	0.20
	East Asian combined	5,664	18,082			5.16 × 10^−16^	1.20 (1.15–1.26)	0.42
Caucasian	USA	1,360	7,952	0.806	0.780	5.71 × 10^−3^	1.19 (1.05–1.34)	
	Scandinavia	1,732	1,788	0.801	0.782	5.44 × 10^−2^	1.12 (1.00–1.26)	
	Caucasian combined	3,092	9,740			1.00 × 10^−3^	1.15 (1.06–1.25)	0.49
	All combined	8,756	27,822			3.28 × 10^−18^	1.19 (1.14–1.24)	0.51

RAF: risk allele frequency, CI: confidence interval.

### Sex-stratified association

AIS has an ample clinical evidence of sexual dimorphism^13^. In our previous study, we investigated *BNC2* expression in a variety of human tissues and found that *BNC2* expression is highest in uterus, suggesting its sex-related biological function^6^. Therefore, we performed sex-stratified analyses to determine whether a genetic difference existed between male and female. We could obtain sex information for both cases and controls in five cohorts. We could obtain 6,266 cases and 15,292 controls in the female-only analysis, and 485 cases and 10,490 controls in the male-only analysis (Supplementary Tables 1 and 2**)**. In both sexes, we could not find genome-wide level significant association (*P* = 5 × 10^−8^); particularly in male, the *P* value did not even reach to the nominal association level (*P* = 5 × 10^−2^) (Tables 2 and 3). However, the ORs were similar between male and female, which were similar to that in the analysis disregarding the sex (Table 1).

**Table 2:** Association of rs3904778 with adolescent idiopathic scoliosis in female.

Population	Study	Number of samples		RAF		*P* value	Odds ratio (95% CI)	P_het_
		Case	Control	Case	Control			
Japanese	Japanese 1	2,004	4,757	0.460	0.426	3.75 × 10^−5^	1.18 (1.09–1.27)	
	Japanese 2	905	3,135	0.476	0.417	6.30 × 10^−6^	1.27 (1.15–1.41)	
Chinese	Guangzhou	561	594	0.352	0.356	8.40 × 10^−1^	0.98 (0.83–1.17)	
	Hong Kong	152	192	0.378	0.315	8.30 × 10^−2^	1.32 (0.96–1.81)	
	East Asian combined	3,622	8,678			4.78 × 10^−5^	1.20 (1.10–1.30)	0.08
Caucasian	USA	1,159	4,826	0.807	0.780	5.50 × 10^−3^	1.21 (1.06–1.38)	
	Scandinavia	1,485	1,788	0.800	0.782	7.31 × 10^−2^	1.12 (0.99–1.26)	
	Caucasian combined	2,644	6,614			1.50 × 10^−4^	1.16 (1.06–1.26)	
	All combined	6,266	15,292			2.93 × 10^−7^	1.18 (1.11–1.25)	0.16

RAF: risk allele frequency, CI: confidence interval.

**Table 3:** Association of rs3904778 with adolescent idiopathic scoliosis in male.

Population	Study	Number of samples		RAF		*P* value	Odds ratio (95% CI)	P_het_
		Case	Control	Case	Control			
Japanese	Japanese 1	105	6,383	0.447	0.405	2.42 × 10^−1^	1.18 (0.89–1.19)	
	Japanese 2	50	412	0.480	0.482	9.73 × 10^−1^	0.99 (0.66–1.50)	
Chinese	Guangzhou	98	469	0.367	0.319	1.87 × 10^−1^	1.24 (0.90–1.71)	
	Hong Kong	31	102	0.387	0.289	1.45 × 10^−1^	1.55 (0.86–2.81)	
	East Asian combined	284	7,366			5.62 × 10^−2^	1.19 (1.00–1.43)	0.67
Caucasian	USA	201	3,124	0.798	0.780	5.96 × 10^−1^	1.08 (0.81–1.45)	
	All combined	485	10,490			5.72 × 10^−2^	1.16 (1.00–1.35)	0.76

RAF: risk allele frequency, CI: confidence interval.

### Fine mapping

**Figure 2:**
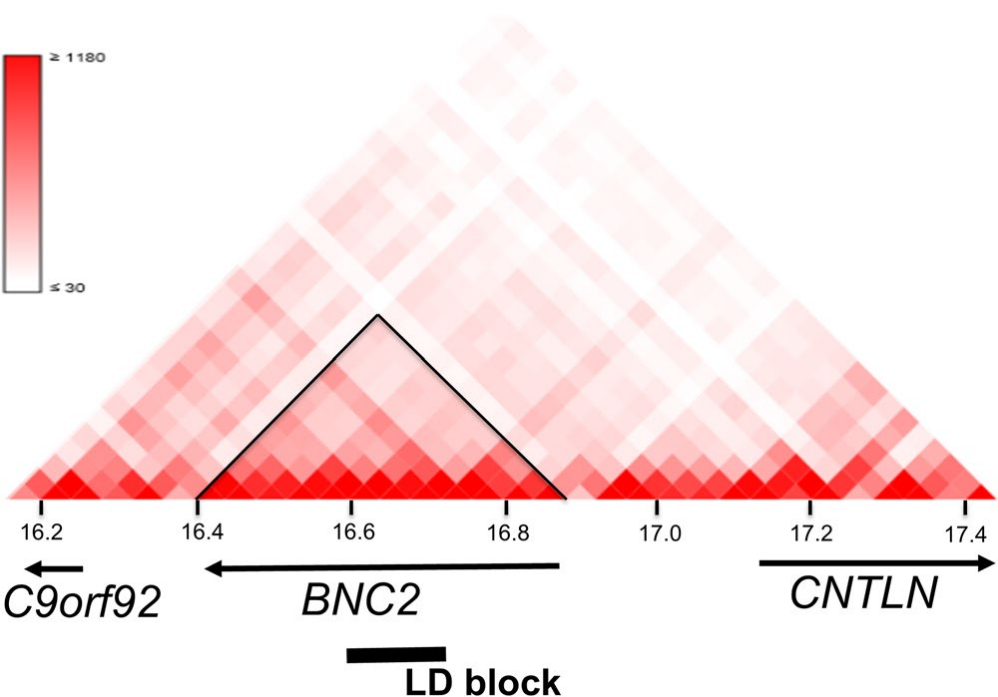
Topologically associated domain around the AIS associated region on chromosome 9p22.2. The Hi-C interaction in H1-mesenchymal stem cell generated by using Interactive Hi-C Data Browser. Only *BNC2* lies within the topologically associated domain (black triangle) that contains the linkage disequilibrium (LD) block of the AIS associated SNPs (bold line). The LD block is contained in *BNC2*.

The landmark SNP rs3904778 is located in intron 3 of *BNC2*, and *BNC2* is the only gene contained within the linkage disequilibrium (LD) block (*r*^2^ > 0.8) represented by rs3904778. The topologically associated domain (TAD) is the partition of the genome that represents a regulatory unit within which enhancers and promoters can interact^14^. To identify the candidate susceptibility gene in the locus, we evaluated the TAD around the associated SNPs using H1-mesenchymal stem cell. Hi-C data^15^ (http://promoter.bx.psu.edu/hi-c/view.php) revealed that *BNC2* was the only gene included in the TAD that contained the LD block of the associated SNPs (Fig. 2). The data strongly suggested that *BNC2* is the most plausible AIS susceptibility gene at this locus.

## Discussion

In the present study, we have performed a meta-analysis for the genetic association of rs3904778 with AIS using more than 36,000 subjects from eight independent multi-ethnic cohorts. To date, no large-scale replication study for the association of the AIS locus has been conducted. Previously, we demonstrated that rs3904778 had significant association with AIS in Japanese and Chinese^6^; however, no evidence has been reported regarding its association in non-East Asian populations. The present study not only gave solid evidence of association of the locus in additional Chinese cohorts, but also revealed that it had significant association in Caucasian, suggesting the global significance of this AIS locus. Previous lack of association in Caucasian may be due to lack of power because the OR of this locus is about 1.2, suggesting relatively large sample size is optimal for identification.

The most significantly associated SNPs are clustered in intron 3 of *BNC2*. *BNC2* is the only gene contained in the LD block of the associated SNPs. TAD containing the LD block only contained *BNC2* (Fig. 2). These genome data strongly suggest that *BNC2* is the AIS susceptibility gene in the locus. rs10738445 in the locus is in high LD (r^2^ = 0.9) with rs3904778. Genevar (Gene Expression Variation) data revealed that the risk allele of the functional SNP in this locus, rs10738445, increased *BNC2* expression (p = 0.048)^6^. Our previous *in vitro* analyses revealed that the risk allele of rs10738445 functioned as an enhancer element and caused increased *BNC2* expression through the increased binding of a transcription factor, YY1 (Ying-Yang 1)^6^. *BNC2* was highly expressed in musculoskeletal tissues such as spinal cord, bone and cartilage^6^. GTEx database also showed similar expression pattern; BNC2 expression was the highest in uterus followed by ovary and nerve. We hypothesized that increased *BNC2* expression in these tissues lead to susceptibility of AIS. Actually, the over-expression of *Bnc2* in zebrafish caused scoliosis-like deformity^6^.

To gain insight into the sex difference in AIS susceptibility, we examined sex-stratified association of rs3904778. While the association was almost genome-wide significant level in the female-only analysis (6,266 cases and 15,292 controls), no significant association was obtained in the male-only analysis (485 cases and 10,490 controls) (Tables 2, 3). This is most probably due to be lack of power in the male analysis; in the analysis, sample size was small, especially in the case group, which reflected the female prevalence in all ethnic populations^6,7,16^. It is of note that the ORs were similar in both sex-stratified analysis. Further analysis with a sufficient sample size will be necessary for the male AIS study, which would inevitably be an international, mutli-center study.

## Methods

### Subjects and genotyping

We obtained informed consent from all subjects and/or their parents. The ethics committee of RIKEN approved this study. All experiments were performed in accordance with relevant guidelines and regulations. The datasets generated during the current study are available from the corresponding authors on reasonable request. AIS subjects were diagnosed through clinical and radiological examinations according to the previously described criteria^4,6,9^. The subjects in the Japanese and Nanjing-Chinese cohorts were recruited and genotyped as previously described^4,6,9^. The detail of beadchip information, quality control and statistical analysis were also previously described^4,6,9^. The details of additional studies (Guangzhou, Hong Kong, Beijing, USA, and Scandinavia studies) were described as below.

#### Guangzhou study

We recruited AIS subjects from the First Affiliated Hospital and Sun Yat-sen Memorial Hospital of Sun Yat-sen University as previously described^12^. We recruited control subjects from individuals who received scoliosis screening at middle and primary schools in Guangzhou and fracture patients selected from the First Affiliated Hospital and Sun Yat-sen Memorial Hospital of Sun Yat-sen University. Orthopedic surgeons evaluated these subjects with Adam’s forward bending test and scoliometers to screen scoliosis. We extracted genomic DNA from blood using DNA Blood Mini-kit (Tiangen Biotech, Beijing, China). The primer extension sequencing (SNaPshot) assay (Applied Biosystems, CA, USA) was used for genotyping and the results were analyzed by GeneMarker software (SoftGenetics LLC, PA, USA) at Beijing Genomics Institute (Shenzhen, China) and checked by visual inspection of I.K. and H.D.

#### Hong Kong study

We recruited AIS subjects from the Duchess of Kent Children’s Hospital in Hong Kong with previously described inclusion criteria^11^. We randomly selected control subjects from the subjects recruited for the Genetic Study of Degenerative Disc Disease project^17^. We confirmed control subjects did not have scoliosis by MRI examination of the spine. We extracted genomic DNA from peripheral blood lymphocytes using standard procedures. We used the PCR-based invader assay (Third Wave Technologies, WI, USA) for genotyping.

#### Beijing Study

We recruited AIS subjects from Peking Union Medical College Hospital. All subjects underwent clinical and radiologic examination and expert spinal surgeons evaluated scoliosis. We extracted genomic DNA from peripheral blood using QIAamp DNA Blood Mini Kit (Qiagen, Hilden, Germany). We used the MassARRAY system (Agena Bioscience, San Diego, CA, USA) for genotyping.

#### USA study

We recruited AIS subjects at Texas Scottish Rite Hospital for Children as previously described^7^ and used the Illumina HumanCoreExome Beadchip array for genotyping. For controls, we utilized a single dataset of individuals downloaded from the dbGaP web site (http://www.ncbi.nlm.nih.gov/sites/entrez?db = gap) from Geisinger Health System-MyCode, eMERGE III Exome Chip Study under phs000957.v1.p1 (https://www.ncbi.nlm.nih.gov/projects/gap/cgi-bin/study.cgi?study_id = phs000957.v1.p1). The dbGaP controls were previously genotyped on the same microarray platform used for cases. Only subjects of self-reported Non-Hispanic White were included in the present study. Phenotypes of all controls were reviewed to exclude subjects having musculoskeletal or neurological disorders. We applied initial per sample quality control measures and excluded sex inconsistencies and any with missing genotype rate per person more than 0.03. Remaining samples were merged using the default mode in PLINK.1.9 (ref.^15^). Duplicated or related individuals were removed as previously described^18^. We used principal component analysis (PCA)^19^ on the merged data projected onto HapMap3 samples to correct possible stratification^20^. After quality controls, 9,312 subjects (1,360 AIS patients and 7,952 controls) were included for the current study. We applied initial per-SNPs quality control measures using PLINK including genotyping call-rate per marker (>95%), minor allele frequency (>0.01) and deviation from Hardy-Weinberg equilibrium (cutoff p-value = 10^−4^). We imputed genotypes for the region around rs3904778 using minimac3^21^ with the 1000G-Phase3.V.5 reference panel accoding to the instructions of the software (http://genome.sph.umich.edu/wiki/Minimac3_Imputation_Cookbook).

#### Scandinavia study

We recruited AIS subjects from six hospitals in Sweden and one in Denmark as with previously described inclusion criteria^22–25^. We recruited control subjects from the Osteoporosis Prospective Risk Assessment cohort and PEAK-25 cohort^26,27^. Dual-energy X-ray absorptiometry (DXA) scan was performed in both cohorts and subjects with any sign of scoliosis on DXA were excluded. We extracted genomic DNA from blood or saliva using the QIAamp 96 DNA Blood Kit and Autopure LS system (Qiagen, Hilden, Germany). We used iPLEX Gold chemistry and MassARRAY system (Agena Bioscience, CA, USA) for genotyping. Two persons checked genotype calls using the MassARRAY Typer v4.0 Software (Agena Bioscience).

### Statistical analysis

The association between rs3904778 and AIS in each study was evaluated by the Cochrane-Armitage trend test aside from the Japanese 1 and USA studies since rs3904778 was an imputated SNP in the two studies. The Japanese 1 study was analyzed as previously described^6^. For the USA study, Mach2dat software^28^ was used to test the imputed allele dosages of rs3904778 by logistic regression with gender and principal components as covariates. Data from the eight studies were combined using the inverse-variance method assuming a fixed-effects model in the METAL software package (http://csg.sph.umich.edu//abecasis/Metal/)^29^. The heterogeneity among studies was tested using Cochran’s Q test based upon inverse variance weights using METAL.

## Electronic supplementary material

Supplementary Info File 1

Supplementary Info File 2
